# Common and Distinct Roles of Juvenile Hormone Signaling Genes in Metamorphosis of Holometabolous and Hemimetabolous Insects

**DOI:** 10.1371/journal.pone.0028728

**Published:** 2011-12-08

**Authors:** Barbora Konopova, Vlastimil Smykal, Marek Jindra

**Affiliations:** 1 Biology Center, Academy of Sciences of the Czech Republic, Ceske Budejovice, Czech Republic; 2 Department of Molecular Biology, University of South Bohemia, Ceske Budejovice, Czech Republic; University of Dayton, United States of America

## Abstract

Insect larvae metamorphose to winged and reproductive adults either directly (hemimetaboly) or through an intermediary pupal stage (holometaboly). In either case juvenile hormone (JH) prevents metamorphosis until a larva has attained an appropriate phase of development. In holometabolous insects, JH acts through its putative receptor Methoprene-tolerant (Met) to regulate *Krüppel-homolog 1* (*Kr-h1*) and *Broad-Complex* (*BR-C*) genes. While Met and Kr-h1 prevent precocious metamorphosis in pre-final larval instars, BR-C specifies the pupal stage. How JH signaling operates in hemimetabolous insects is poorly understood. Here, we compare the function of *Met*, *Kr-h1* and *BR-C* genes in the two types of insects. Using systemic RNAi in the hemimetabolous true bug, *Pyrrhocoris apterus*, we show that Met conveys the JH signal to prevent premature metamorphosis by maintaining high expression of *Kr-h1*. Knockdown of either *Met* or *Kr-h1* (but not of *BR-C*) in penultimate-instar *Pyrrhocoris* larvae causes precocious development of adult color pattern, wings and genitalia. A natural fall of *Kr-h1* expression in the last larval instar normally permits adult development, and treatment with an exogenous JH mimic methoprene at this time requires both *Met* and *Kr-h1* to block the adult program and induce an extra larval instar. Met and Kr-h1 therefore serve as JH-dependent repressors of deleterious precocious metamorphic changes in both hemimetabolous and holometabolous juveniles, whereas BR-C has been recruited for a new role in specifying the holometabolous pupa. These results show that despite considerable evolutionary distance, insects with diverse developmental strategies employ a common-core JH signaling pathway to commit to adult morphogenesis.

## Introduction

Winged insects have evolved diverse modes of metamorphosis [1]. Hemimetabolous insects such as grasshoppers, true bugs or cockroaches develop from larvae (also called nymphs) that resemble adults, possess externally growing wing pads, and metamorphose during the final molt by acquiring perfect wings and genitalia. In contrast, larvae of holometabolous insects including flies, butterflies or beetles can differ dramatically from the adults. They undergo a two-stage “complete” metamorphosis (holometaboly), first forming an intermediate called the pupa before changing into a winged adult. Holometaboly has evolved from hemimetaboly and, judging by the number of known species, has become the most successful developmental strategy on land [2].

In both hemimetabolous and holometabolous insects, the developmental switch between juvenile and adult forms depends on juvenile hormone (JH), a sesquiterpenoid produced by the corpora allata gland [3]. The presence of JH in pre-final larval instars ensures that the next molt, promoted by ecdysteroids, produces another, only a larger larva [4], [5]. At an appropriate stage, a natural drop of JH secretion permits metamorphosis. Experimental removal of JH at earlier times activates the metamorphic program prematurely, whereas supply of ectopic JH to final-instar larvae or pupae causes repetition of larval or pupal instars, respectively [6]–[8].

Although the anti-metamorphic effect of JH was discovered in the hemimetabolous true bug, *Rhodnius prolixus*, [9], [10], our knowledge on the molecular mode of JH action almost exclusively derives from studies in holometabolans. JH signals through its putative intracellular receptor, the bHLH-PAS protein Methoprene-tolerant (Met), originally identified in the fruit fly *Drosophila melanogaster* [11], [12]. In the red flour beetle, *Tribolium castaneum*, loss of Met triggers pupation of larvae during pre-final instars [13] – a classic precocious metamorphosis phenotype caused by deficiency of JH itself [14]. In response to JH, Met regulates expression of transcription factor genes *Krüppel-homolog 1* (*Kr-h1*) and *Broad-Complex* (*BR-C*) [15]–[18], and loss of *Kr-h1* also elicits precocious metamorphosis of beetle larvae [17]. *BR-C* is dispensable in holometabolous larvae until the onset of metamorphosis, when it specifies pupal features [16], [19]–[23]. Upon pupation both *BR-C* and *Kr-h1* are naturally down-regulated by the absence of JH to allow adult development [16]–[18], [20], [22]. Of the three JH-signaling genes, *BR-C* has been functionally studied in hemimetabolous insects, where, unlike in holometabolans, it is required for development of the embryonic germ band [24], [25] and for anisometric growth of the larval wing pads [26].

To provide a direct comparison of JH signaling in holometaboly and hemimetaboly, we have examined the function of *Met*, *Kr-h1* and *BR-C* in the hemimetabolous firebug, *Pyrrhocoris apterus* (true bugs, Hemiptera). We show that despite the diverse developmental strategies and the vast evolutionary distance between them, transduction of the anti-metamorphic JH signal relies on the common-core elements, Met and Kr-h1. In both insect types, Kr-h1 acts as a strictly JH- and Met-dependent repressor of metamorphosis. In contrast, the function of BR-C has changed from promoting progressive development of hemimetabolous larvae to a new role in specifying the holometabolous pupa.

## Results

### JH Signaling Genes Are Conserved in Insects with Diverse Types of Development

As the first step towards functional comparison between holometaboly and hemimetaboly, we have isolated cDNAs encoding the putative JH receptor Met (JN416984) and its target genes *Kr-h1* (JN416987) and *BR-C* (JN416990), from *Pyrrhocoris apterus*, and *Met* (JN416985) and *Kr-h1* (JN416988) cDNAs from another true bug, *Rhodnius prolixus*. Alignments amongst the orthologs reveal conservation of the main functional domains (Fig. S1), namely the basic helix-loop-helix (bHLH) region and two Per-Arnt-Sim (PAS) domains in Met, eight zinc-finger motifs in Kr-h1, and a Broad-Tramtrac-Bric-a-brac (BTB) domain followed by one of the alternative zinc-finger isoforms (Z2) in BR-C. Therefore, the three JH signaling genes are common to insect orders developing through holometaboly and hemimetaboly. In fact, their conservation predates the origin of metamorphosis, as we have found *Met* (JN416986), *Kr-h1* (JN416989) and *BR-C* (JN416991) orthologs in the firebrat, *Thermobia domestica*, a representative of the primitive lineage of non-metamorphosing wingless insects (Fig. S1).

### Developmental Regulation of the JH-Response Genes in *Pyrrhocoris*

The firebug invariantly undergoes four larval molts demarcating five larval instars (L1–L5), followed by a metamorphic molt, which produces an adult possessing external genitals and wings with a specific color pattern. Maintenance of the larval state in *Pyrrhocoris* requires JH, as has been demonstrated by removal of the JH-producing corpora allata gland (allatectomy) from penultimate (L4) larvae [27]. Conversely, metamorphosis is permitted by a natural decline in JH titer during the last larval instar (L5), as supplying L5 larvae with JH mimics induces an extra larval stage (reference [28] and this work).

**Figure 1:**
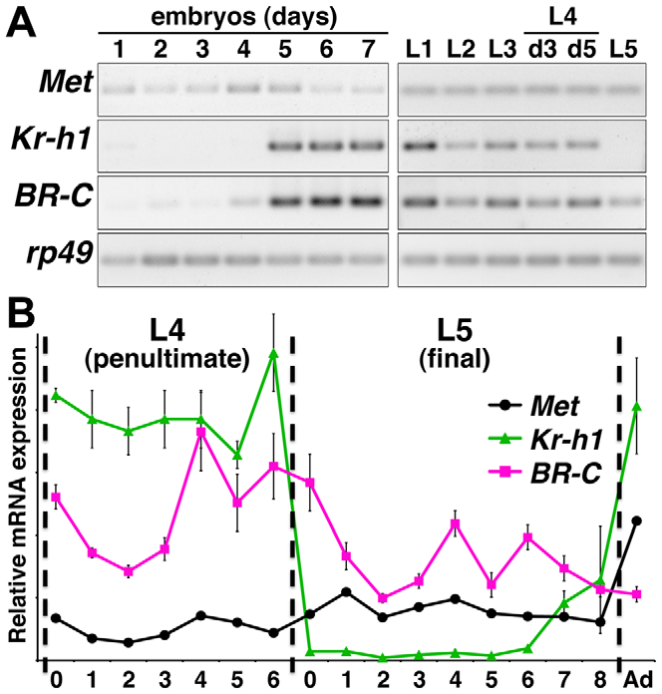
Developmental expression of *Pyrrhocoris* JH signaling genes. **A**) Semi-quantitative RT-PCR on total RNA from embryos (26 temperature cycles) or larvae (28 cycles) of indicated stages. d3, d5, mid- and late-L4 instar larvae, respectively. **B**) Relative mRNA levels on successive days of larval development were assessed with qRT-PCR and normalized to *rp49* mRNA. Dashed lines mark ecdyses to L4, L5 and adult stages. Values are mean ± SD from three measurements on RNA isolated from individual animals.

We examined how expression of *Met*, *Kr-h1* and *BR-C* during *Pyrrhocoris* development correlates with this regulation by JH. From embryogenesis to adulthood, *Met* mRNA persisted without major fluctuations through all larval stages (Fig. 1). Such a constitutive presence agrees with the presumed JH receptor role of Met and with the ability of L5 larvae that naturally lack JH to respond to exogenous JH mimics by forming an extra larval instar. Temporal profiles of *Kr-h1* and *BR-C* were more dynamic. Both transcripts reached their highest levels during the second half of embryogenesis, then *BR-C* oscillated throughout larval development, whereas *Kr-h1* was not detected in last-instar larvae, L5 (Fig. 1A). A more detailed profile revealed that upon ecdysis to L5, *BR-C* mRNA decreased but remained expressed to adulthood, while *Kr-h1* expression plummeted from its L4 level by more than 50-fold and was virtually undetectable for the first six days of L5 until the next rise, likely induced by a new surge of JH in pharate adults (Fig. 1B). Allatectomy of adult *Pyrrhocoris* females drastically reduced *Kr-h1* mRNA (494-fold, *n* = 3), confirming that *Kr-h1* transcription absolutely requires the natural source of JH. The expression pattern suggested that Kr-h1 prevents metamorphosis until the final larval instar, when the Kr-h1-free (and JH-free) period may be necessary to initiate metamorphosis.

### Kr-h1 Is a Met-Dependent Repressor of Precocious Adult Development in *Pyrrhocoris* Larvae

**Figure 2:**
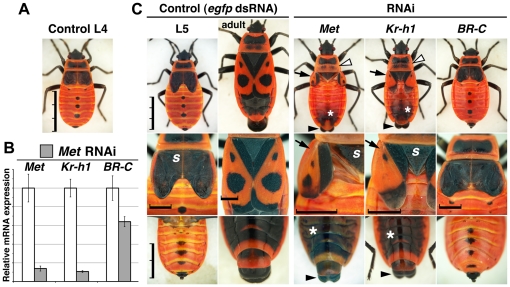
Loss of *Met* or *Kr-h1* causes precocious metamorphosis in *Pyrrhocoris* larvae. **A**) Larvae newly ecdysed to the L4 instar were injected with control (*egfp*) or *Met* dsRNA. **B**) Efficacy of *Met* mRNA depletion and its effect on expression of *Kr-h1* and *BR-C* expression were determined by qRT-PCR three days after injection of *Met* dsRNA (gray columns) relative to *egfp* dsRNA controls (open columns) arbitrarily set to 100%. Values are mean ± SD from *n* = 5 animals. **C**) *Met*, *Kr-h1* and *BR-C* RNAi phenotypes after ecdysis to the L5 stage as compared to control L5 larvae and adults (left two columns). Animals in the top row are to the same scale; the middle and bottom rows show details of wings and of the ventral abdomen, respectively. Precocious adult attributes upon *Met* and *Kr-h1* RNAi include color-patterned articulated wings (arrows) separated from the scutellum (*s*), extended notum (open arrowheads) with two posterior black spots, external male genitals (solid arrowheads), and dark abdominal cuticle (asterisks). Compared to control L5, *BR-C(RNAi)* larvae display retarded wing growth but no precocious adult development. For quantitative data see Table 1. (Scale bars: A and C, top row, 3 mm; C, middle row, 1 mm; C, bottom row, 2 mm).

To test whether Met and Kr-h1 are indeed required to maintain the juvenile character of hemimetabolous larvae, we utilized systemic RNAi in *Pyrrhocoris*. Injection of *Met* dsRNA into early-L4 larvae caused an 86% knockdown of *Met* mRNA (Fig. 2B), followed by precocious appearance of adult attributes after the ensuing molt, which would have otherwise produced an L5 larva (Fig. 2C and Table 1). Despite their smaller body size compared to normal adults, *Met(RNAi)* males had external genitals (Fig. 2C). Instead of the solid black and fully attached L5 wing pads, these adultoids developed small but movable, articulated wings, liberated from the scutellum and the tergites. The black melanin pigment disappeared from specific regions of these wings to leave black spots and edges on the red background, a pattern only seen in adults (Fig. 2C). On the thorax, the notum expanded posteriorly and also displayed an adult-specific color pattern. Like in adults, abdominal cuticle tanned with melanin. We did not determine whether particular regions of the cuticle, produced by individual epidermal cells, were purely adult or whether they had a mixed larval-adult identity, so this intriguing possibility remains [29]. Unable to molt again, all L5 adultoids were terminally arrested.

**Table 1:** Loss of *Met* or *Kr-h1* triggers precocious adult development in *Pyrrhocoris* larvae.

dsRNA	n	L4 Death^a^	Abnormal L5 phenotypes		Normal adults
			Adult hallmarks^b^	Short wings	
***Met***	29	3	26	0	0
***Kr-h1***	31	3	28	0	0
***BR-C***	34	15	0	19	0
**control**	12	1	0	0	11

a Lethality without a specific external phenotype.

b Precocious metamorphosis.

*Kr-h1* mRNA levels were reduced by 90% in L4 larvae subjected to *Met* RNAi (Fig. 2B), demonstrating that *Kr-h1* in *Pyrrhocoris* was a Met-dependent target gene as in *Tribolium* [15], [17]. This reduction paralleled the natural fall of *Kr-h1* expression upon ecdysis to L5 (Fig. 1B), suggesting that the normal function of Met during the L4 stage is to respond to endogenous JH by maintaining high expression of *Kr-h1*, which in turn prevents adult development. Consistent with this prediction, larvae injected with *Kr-h1* dsRNA at the L4 stage formed L5 adultoids similar to *Met(RNAi)* animals (Fig. 2C and Table 1). Occasionally their wings grew larger than upon *Met* RNAi, and some individuals only showed part of the adult color pattern (Fig. 2C). Clearly, the loss-of-function phenotypes of both genes were in good concert and demonstrated precocious metamorphosis of *Pyrrhocoris* larvae.

**Figure 3:**
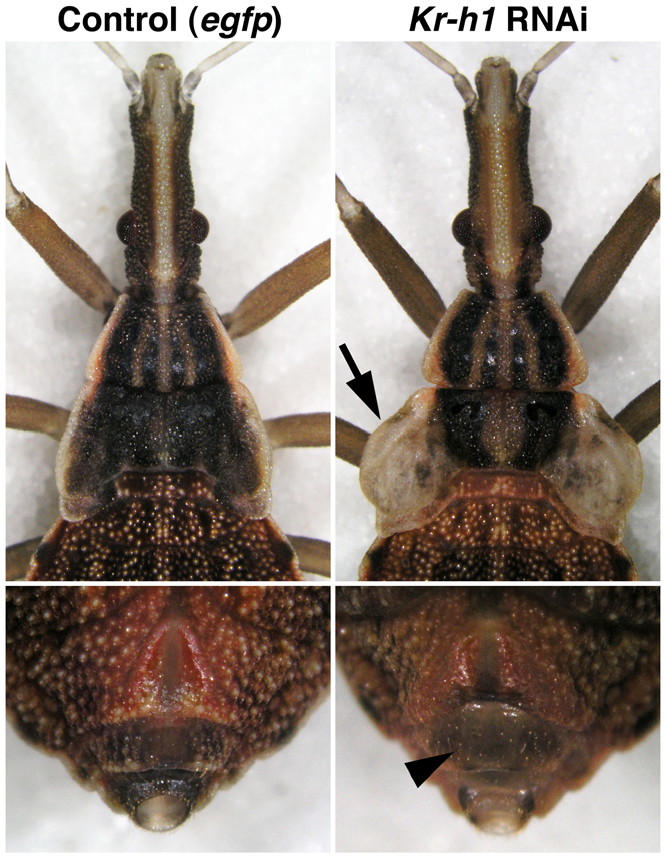
Precocious development of adult features after *Kr-h1* RNAi in the blood sucking bug, *Rhodnius prolixus*. When subjected to *Kr-h1* dsRNA injection as L3 larvae, the animals displayed abnormal growth and venation of wing lobes after molting to the L4 stage (right) as compared to control L4 larvae. We also noticed premature development of external genitals in *Kr-h1(RNAi)* animals (arrowhead).

To verify the function of *Kr-h1* in an independent system, we performed RNAi in the blood-sucking bug *Rhodnius*, the very model in which juvenile hormone had been postulated nearly eight decades ago [9], [10]. Upon molting to the L4 penultimate instar, all *Rhodnius* larvae (*n* = 20) injected with *Kr-h1* dsRNA at the previous L3 instar showed accelerated growth of wings and genitals (Fig. 3), both typical hallmarks of adult morphogenesis.

In contrast to *Met* and *Kr-h1*, injection of *BR-C* dsRNA into *Pyrrhocoris* L4 larvae produced no signs of premature adult development (Fig. 2C and Table 1). *BR-C* RNAi administered early during the L4 instar reduced *BR-C* mRNA levels to 17.7±2.0% (*n* = 5 animals) and caused either lethality at the end of the L4 instar or compromised growth of the wing pads in animals that successfully molted to the L5 instar (Table 1). A similar wing defect was observed after ecdysis to the L4 instar in 100% of animals (*n* = 22) that had been given *BR-C* dsRNA as L3 larvae. The retarded wing growth was previously shown for *BR-C* RNAi in the milkweed bug, *Oncopeltus fasciatus* [26]. When compared to *Kr-h1*, *BR-C* mRNA expression was neither as strongly dependent on Met at the L4 instar (Fig. 2B) nor did it completely cease upon ecdysis to L5 (Fig. 1B). Therefore, Kr-h1 but not BR-C functions as a JH- and Met-dependent repressor of hemimetabolous metamorphosis.

### Met and Kr-h1 Mediate the Anti-Metamorphic Effect of Methoprene

**Figure 4:**
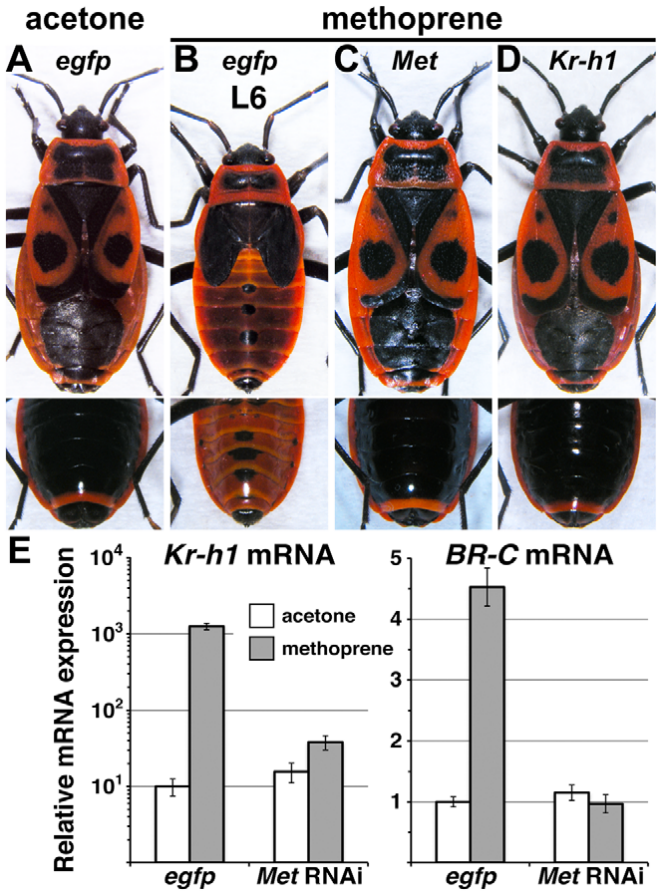
*Met* and *Kr-h1* mediate the anti-metamorphic effect of exogenous JH mimic. **A–D**) Animals received either control (*egfp*), *Met* or *Kr-h1* dsRNA as late-L4 larvae, followed by mock (acetone) or JH mimic (methoprene) treatment early at the L5 instar. Bottom row shows ventral view of the abdomens. Acetone-treated larvae produced normal adults (A). Methoprene induced a supernumerary larval instar (L6) whose wing pads remained black and attached to the tergites, while the abdominal cuticle tanned only partly (B). Knockdown of *Met* (C) or *Kr-h1* (D) prior to methoprene treatment restored normal adult development. **E**) Ectopic Met-dependent induction of *Kr-h1* and *BR-C* by methoprene at the L5 instar. Relative mRNA levels of *Kr-h1* and *BR-C* in the abdominal epidermis of animals injected with control (*egfp*) or *Met* dsRNA and subjected to hormonal treatment as described above were assessed on day 4 of the L5 instar. Values are mean ± SD from *n* = 5 animals. Note that the much higher *Kr-h1* induction (left) is on the logarithmic scale.

When treated with JH mimics, final instar *Pyrrhocoris* larvae fail to metamorphose to adults and instead repeat the larval program in the succeeding supernumerary “L6” instar [28]. We achieved the same effect by topical treatment of early-L5 larvae with the JH mimic methoprene (Fig. 4A,B) in 100% (*n* = 18) of animals. In this background we then tested whether *Met* and *Kr-h1* mediated the response to the exogenous JH activity.

When *Met* was silenced by dsRNA injection at the end of the L4 instar, the following application of methoprene no longer perturbed adult development in 96% (*n* = 23) of treated animals, and these rescued adults were externally indistinguishable from controls receiving no methoprene (Fig. 4A,C). The “status-quo” effect of methoprene was likewise prevented by *Kr-h1* RNAi (Fig. 4D) in 12 out of 13 animals. Therefore, both *Met* and its target *Kr-h1* were necessary for the capacity of methoprene to block metamorphosis and induce the L6 larva. In addition, neither *Met* nor *Kr-h1* appeared to be required for the normal L5-to-adult transition, since dsRNA injection to late-L4 larvae, as opposed to RNAi applied early during the L4 stage, did not interfere with metamorphosis. This result corresponded with the natural absence of *Kr-h1* expression during most of the final larval instar (Fig. 1B).

The above data indicated that the Met-dependent reiteration of the larval program, induced by methoprene, occurred via ectopic transcriptional re-activation of *Kr-h1* during the L5 instar. Indeed, levels of *Kr-h1* mRNA in the epidermis (or in the whole body, data not shown) of mid-L5 larvae, which had been treated with methoprene as L4, exceeded levels normally observed at the L5 instar by 125-fold (Fig. 4E). This induction was the opposite to the natural drop of *Kr-h1* expression upon L4 to L5 ecdysis (Fig. 1B), and it was abolished by *Met* RNAi (Fig. 4E). By contrast, *BR-C* mRNA was only induced 4.5-fold with methoprene, again in a Met-dependent manner (Fig. 4E). The striking difference in the fold mRNA induction between *Kr-h1* and *BR-C* did not result from difference in the mRNA levels induced by methoprene but from the extremely low expression of *Kr-h1* in mid-L5 larvae (Fig. 1).

## Discussion

**Figure 5:**
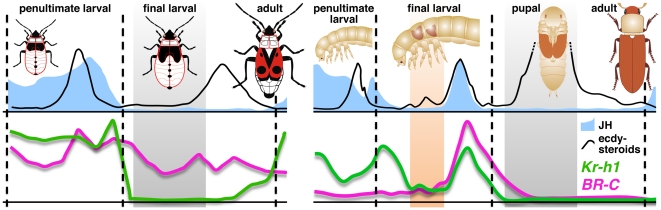
Regulation of hemimetaboly and holometaboly. *Pyrrhocoris* (left) and *Tribolium* (right) cartoons signify the main innovations – postponement of wing development and the resting pupal stage in holometabolans. The absence of JH-dependent *Kr-h1* expression in pupae and final instar hemimetabolous larvae (gray shaded areas) is prerequisite to adult development in both types of metamorphosis, supporting the view that these final juvenile stages of both insects types may be homologous [1], [35]. The orange shaded area marks a period of low *Kr-h1* activity in the absence of JH, which is necessary to permit partial metamorphosis during the pupal molt, specified by the newly acquired function of *BR-C* in holometabolans. Gene expression profiles for *Pyrrhocoris* and *Tribolium* are from Figure 1B and from [17], respectively. JH and ecdysteroid titers are from *Blattella germanica* [34] (left) and *Manduca sexta* [5] (right).

The likely JH receptor Met and its targets *Kr-h1* and *BR-C* play key roles during holometabolous insect metamorphosis. Our present data afford a direct comparison with how the three genes function in the ancestral hemimetabolous development (Fig. 5). We provide three lines of evidence that the JH-free and Kr-h1-free period in the final larval instar (L5) of the *Pyrrhocoris* bug is critical for adult transition: (*i*) administration of methoprene to L5 larvae induces Met-dependent *Kr-h1* expression and an extra larval stage, (*ii*) this phenotype is averted if *Met* or *Kr-h1* are silenced prior to methoprene treatment, and (*iii*) premature suppression of *Kr-h1*, either direct or through depletion of Met, triggers precocious adult development. Therefore, the JH/Met-dependent *Kr-h1* activity ensures the larval program, and only when JH disappears from the blood in the last larval instar, transcription of *Kr-h1* ceases for six days to create an opportunity for adult transition (Figs. 1B and 5). Interestingly, the same-length time window for adult commitment was previously defined as a methoprene-sensitive period during the final larval instar of the *Rhodnius* bug [30]. *Kr-h1* now provides a molecular determinant of that window.

Similar regulation applies to holometaboly. In pre-terminal instars of *Tribolium* larvae, *Met* and *Kr-h1* respond to JH by blocking precocious metamorphosis [13], [17]. By mid-final larval instar, expression of *Kr-h1* declines to reappear at the pupal molt, this time together with *BR-C* mRNA (Fig. 5). While the transient down-regulation of *Kr-h1* permits partial metamorphosis (pupation), its co-expression with *BR-C* likely ensures that development does not go too far. This is suggested by appearance of not only pupal but also of adult features in *Tribolium* larvae subjected to *Kr-h1* or *BR-C* RNAi [16], [17], [22], [23]. After the pupal program has been installed, a JH-free period ensures that both *Kr-h1* and *BR-C*, acting downstream of *Kr-h1* in this context [17], [18], are shut down in order for adult morphogenesis to take place (Fig. 5). Giving exogenous JH or its mimics to pupae will re-activate both genes and block adult development [16]–[18], [20].

While the function of *Kr-h1* as a JH-induced repressor of adult morphogenesis is clearly a common trait of holometaboly and hemimetaboly, the role of *BR-C* is not. Studies on the hemimetabolan *Oncopeltus* bug have revealed *BR-C* requirement during embryogenesis and for the anisometric growth of larval wing pads but no *BR-C* expression or function connected with metamorphosis [24], [26]. Conversely, *BR-C* is essential for pupal development but not at earlier stages in representatives of four holometabolan insect orders [16], [19]–[22]. The delay of *BR-C* activity until the pupal stage in Holometabola has been ascribed to an early surge of JH during embryogenesis, which is thought to preserve the seemingly undeveloped (“embryonic”) nature of holometabolous larvae [24], [31]–[33]. However, while *BR-C* is necessary for hemimetabolous embryogenesis [24], [25], its function has not been causally linked with JH in insect embryos.

Unlike in *Oncopeltus*, *BR-C* expression continues, albeit at a lower rate, throughout the last larval instar of *Pyrrhocoris* or another hemimetabolan, the cockroach *Blattella germanica*, in the absence of JH [34] (Fig. 5). Consistent with this pattern, our data show that compared to *Kr-h1*, expression of *BR-C* much less depends on JH and that in contrast to *Kr-h1* or *Met*, removal of *BR-C* cannot accelerate metamorphosis in bug larvae. The changed need for *BR-C* function from hemimetabolous embryos and larvae to holometabolous metamorphosis suggests that during the evolution of holometaboly, *BR-C* has been recruited for the new function in specifying the pupal state.

Kr-h1 is intimately regulated by JH and Met to safeguard juveniles of both hemimetabolous and holometabolous insects against precocious and hence fatal metamorphic changes. Therefore, regardless of the disparate life histories, insects undergoing both types of metamorphosis use a common signaling pathway to commit to adult development. The parallel timing of the critical down-regulation of *Kr-h1* in the final-instar *Pyrrhocoris* larva and in the holometabolous pupa (Fig. 5) supports the view that these stages represent ontogenetically homologous units [1], [35], rather than hypotheses building on the assumption that the pupa has originated via compression of all hemimetabolous larval instars into one [24], [32], [33].

## Materials and Methods

### Insects

*Pyrrhocoris apterus* (short-winged form) was maintained at 25°C and a photoperiod of 18 h light to 6 h dark, on dry linden seeds and was supplemented with water. Eggs were collected daily and larvae of particular instars were identified based on the size of the body and wing pads; staging within instars relied on measuring time after ecdysis.

### cDNA Cloning

Partial sequences for *Pyrrhocoris Met*, *Kr-h1* and *BR-C* genes were isolated by using touch-down nested RT-PCR with degenerate primers (Table S1), mapping to conserved domains. cDNA ends of selected genes were amplified with the GeneRacer Kit (Invitrogen, Carlsbad, CA).

### mRNA Expression Analysis

Total RNA was isolated from *Pyrrhocoris* embryos, whole larvae or abdominal epidermis with the TRIzol reagent (Invitrogen, Carlsbad, CA). After TURBO DNase (Ambion, Austin, TX) treatment, 2 µg of RNA were used for cDNA synthesis with Superscript II reverse transcriptase (Invitrogen). Relative transcript levels were measured by quantitative RT-PCR using the iQ SYBR Green Supermix kit and the C1000 Thermal Cycler (both from Bio-Rad Laboratories, Hercules, CA). All data were normalized to the relative levels of ribosomal protein (Rp49) mRNA as described [36]. Primer sequences used for qRT-PCR are listed in Table S2.

### RNAi and Methoprene Treatments

dsRNAs comprising 952 bp (*Met*), 844 bp (*Kr-h1*) and 1026 bp (*BR-C*) of the *Pyrrhocoris* cDNA sequences and control dsRNAs encoding the EGFP and MalE proteins (720 bp and 901 bp, respectively) were synthesized by using the T3 and T7 MEGAscript kit (Ambion, Austin, TX). Approximately 2–5 µg of dsRNA (depending on the size of larvae) were injected into the abdomen of CO_2_-anesthesized bugs. For JH mimic treatment, late-L4 stage *Pyrrhocoris* larvae were injected with dsRNA and within 2–3 hours after ecdysis to the L5 instar, a 4-µl drop of acetone-diluted 0.3 mM methoprene (VUOS, Pardubice, Czech Republic) or acetone alone (control) was applied on their dorsal side.

## Supporting Information

Figure S1 **Conservation of JH signaling genes.** Alignments of Met (*A*), Kr-h1 (*B*) and BR-C (*C*) protein sequences from insects representing diverse developmental strategies. Holometaboly: the fruit fly *Drosophila melanogaster*, mosquitoes *Aedes aegypti* and *Culex quinquefasciatus* (Diptera); the silk moth *Bombyx mori* (Lepidoptera); the flour beetle *Tribolium castaneum* (Coleoptera); the honey bee *Apis melifera* (Hymenoptera); the lacewing *Chrysopa perla* (Neuroptera). Hemimetaboly: true bugs *Pyrrhocoris apterus, Rhodnius prolixus* and *Oncopeltus fasciatus* (Hemiptera); the cockroach *Blattella germanica* (Blattodea); the louse *Pediculus humanus corporis* (Phthiraptera). The thrips, *Frankliniella occidentalis* (Thysanoptera), represents neometaboly, an aberrant type of hemimetaboly with multiple resting stages. The firebrat, *Thermobia domestica* (Zygentoma), represents a basal lineage of wingless insects without metamorphosis (ametaboly). *Dm*_Met, *Dm*_Gce, and *Bm*_Met1, *Bm*_Met2 are products of paralogous *Met* genes that have duplicated independently in the *Drosophila* and *Bombyx* lineages, respectively. Database accession numbers for the aligned Met proteins (*A*) are NP_511126.2 (*Drosophila melanogaster* Met), NP_511160.1 (*Drosophila melanogaster* Gce), XP_001660262.1 (*Aedes aegypti*), BAJ05085.1 (*Bombyx mori* Met1), BAJ05086.1 (*Bombyx mori* Met2), ABR25244.1 (*Tribolium castaneum*), XP_395005.3 (*Apis melifera*), JN416984 (*Pyrrhocoris apterus*), JN416985 (*Rhodnius prolixus*), XP_002430841.1 (*Pediculus humanus corporis*), and JN416986 (*Thermobia domestica*). Accession numbers for the Kr-h1 proteins (*B*) are CAA06544.2 (*Drosophila melanogaster*), XP_001863529.1 (*Culex quinquefasciatus*), NP_001129235.1 (*Tribolium castaneum*), NP_001011566.1 (*Apis melifera*), BAJ41258.1 (*Frankliniella occidentalis*), JN416987 (*Pyrrhocoris apterus*), JN416988 (*Rhodnius prolixus*), XP_002428656.1 (*Pediculus humanus corporis*), and JN416989 (*Thermobia domestica*). Accession numbers for the BR-C proteins (*C*) are CAA38476.1 (*Drosophila melanogaster*), AAS80327.1 (*Aedes aegypti*), BAD23979.1 (*Bombyx mori*), joined ABW91135.1 and ABW91137.1 (*Tribolium castaneum*), ABW91140.1 (*Chrysopa perla*), BAJ41241.1 (*Frankliniella occidentalis*), JN416990 (*Pyrrhocoris apterus*), ABA02191.1 (*Oncopeltus fasciatus*), CBJ05858.1 (*Blattella germanica*), and joined GQ983556.1 and JN416991 (*Thermobia domestica*).
(PDF)

Table S1 **Degenerate primers for isolation of** ***Met*** **,** ***Kr-h1*** **and** ***BR-C*** **cDNAs from** ***Pyrrhocoris apterus*** **,** ***Rhodnius prolixus*** **and** ***Thermobia domestica*** **.**
(PDF)

Table S2 **Primers for RT-PCR expression analysis of** ***Pyrrhocoris apterus Met*** **,** ***Kr-h1*** **and** ***BR-C*** **mRNAs.**
(PDF)
