# Supplementary material for: Common and Distinct Roles of Juvenile Hormone Signaling Genes in Metamorphosis of Holometabolous and Hemimetabolous Insects
Source: PLoS One. 2011 Dec 8;6(12):e28728. doi: 10.1371/journal.pone.0028728 (PMC3234286; doi:10.1371/journal.pone.0028728)
Supplement: Table S1 — Degenerate primers for isolation of Met , Kr-h1 and BR-C cDNAs from Pyrrhocoris apterus , Rhodnius prolixus and Thermobia domestica . (PDF) [file pone.0028728.s002.pdf]

Table S1. Degenerate primers for isolation of *Met*, *Kr-h1* and *BR-C* cDNAs from *Pyrrhocoris apterus*, *Rhodnius prolixus* and *Thermobia domestica*.

**Target sequence**	**Forward primer (5'-3')**	**Reverse primer (5'-3')**
Met	GARATGMGIAAYHKIGCNGARAA	ACIARIGTRTTIAYRCANAYRAA
Met, nested	AARCARMGIMGISAIAARYTNAA	GCIAYIATIACCCANCKNACRTC
Kr-h1	CAYTAYMGIACICAYACNGGNGA	TTIARYTGYTTISWRCAIGTRAANCC
Kr-h1, nested	GTICAYMGIMGIATHCAYACNAARG	CAIAYRTAIGGYTTYTCICCNGTRTG
BR-C_Z2	TTYTGYYTIMGNTGGAAYAAYTAYC	TTRTGRTAIGTRTAIATRTGNGTCAT
BR-C_Z2, nested	TTYGARAAYYTIMGIGAYGAYGARG	CKISWRCARTAIACICKYTCRCA
