# Supplementary material for: Common and Distinct Roles of Juvenile Hormone Signaling Genes in Metamorphosis of Holometabolous and Hemimetabolous Insects
Source: PLoS One. 2011 Dec 8;6(12):e28728. doi: 10.1371/journal.pone.0028728 (PMC3234286; doi:10.1371/journal.pone.0028728)
Supplement: Table S2 — Primers for RT-PCR expression analysis of Pyrrhocoris apterus Met , Kr-h1 and BR-C mRNAs. (PDF) [file pone.0028728.s003.pdf]

Table S2. Primers for RT-PCR expression analysis of *Pyrrhocoris apterus Met*, *Kr-h1* and *BR-C* mRNAs.

Target sequence	Forward primer (5'-3')	Reverse primer (5'-3')
Met	TTCTGATGATGGTGAAAAGATG	TATCGCCCCTGACTACTTGG
Kr-h1	GAACGTCTTGTTACACACACC	CCCTACCAGTGTAACTTTTGC
BR-C	TCTCCAAGATGTCATGTTTGAAG	AACCTCGACCGTCTGCCAAAC
*rp49**	CCGATATGTAAAACTGAGGAGAAAC	GGAGCATGTGCCTGGTCTTTT

*Levels of the ribosomal protein *rp49* mRNA were used for normalization of all expression data.
