# Supplementary material for: Common and Distinct Roles of Juvenile Hormone Signaling Genes in Metamorphosis of Holometabolous and Hemimetabolous Insects
Source: PLoS One. 2011 Dec 8;6(12):e28728. doi: 10.1371/journal.pone.0028728 (PMC3234286; doi:10.1371/journal.pone.0028728)
Supplement: Figure S1 — Conservation of JH signaling genes. Alignments of Met (A), Kr-h1 (B) and BR-C (C) protein sequences from insects representing diverse developmental strategies. Holometaboly: the fruit fly Drosophila melanogaster, mosquitoes Aedes aegypti and Culex quinquefasciatus (Diptera); the silk moth Bombyx mori (Lepidoptera); the flour beetle Tribolium castaneum (Coleoptera); the honey bee Apis melifera (Hymenoptera); the lacewing Chrysopa perla (Neuroptera). Hemimetaboly: true bugs Pyrrhocoris apterus, Rhodnius prolixus and Oncopeltus fasciatus (Hemiptera); the cockroach Blattella germanica (Blattodea); the louse Pediculus humanus corporis (Phthiraptera). The thrips, Frankliniella occidentalis (Thysanoptera), represents neometaboly, an aberrant type of hemimetaboly with multiple resting stages. The firebrat, Thermobia domestica (Zygentoma), represents a basal lineage of wingless insects without metamorphosis (ametaboly). Dm_Met, Dm_Gce, and Bm_Met1, Bm_Met2 are products of paralogous Met genes that have duplicated independently in the Drosophila and Bombyx lineages, respectively. Database accession numbers for the aligned Met proteins (A) are NP_511126.2 (Drosophila melanogaster Met), NP_511160.1 (Drosophila melanogaster Gce), XP_001660262.1 (Aedes aegypti), BAJ05085.1 (Bombyx mori Met1), BAJ05086.1 (Bombyx mori Met2), ABR25244.1 (Tribolium castaneum), XP_395005.3 (Apis melifera), JN416984 (Pyrrhocoris apterus), JN416985 (Rhodnius prolixus), XP_002430841.1 (Pediculus humanus corporis), and JN416986 (Thermobia domestica). Accession numbers for the Kr-h1 proteins (B) are CAA06544.2 (Drosophila melanogaster), XP_001863529.1 (Culex quinquefasciatus), NP_001129235.1 (Tribolium castaneum), NP_001011566.1 (Apis melifera), BAJ41258.1 (Frankliniella occidentalis), JN416987 (Pyrrhocoris apterus), JN416988 (Rhodnius prolixus), XP_002428656.1 (Pediculus humanus corporis), and JN416989 (Thermobia domestica). Accession numbers for the BR-C proteins (C) are CAA38476.1 (Drosophila melanogast [file pone.0028728.s001.pdf]

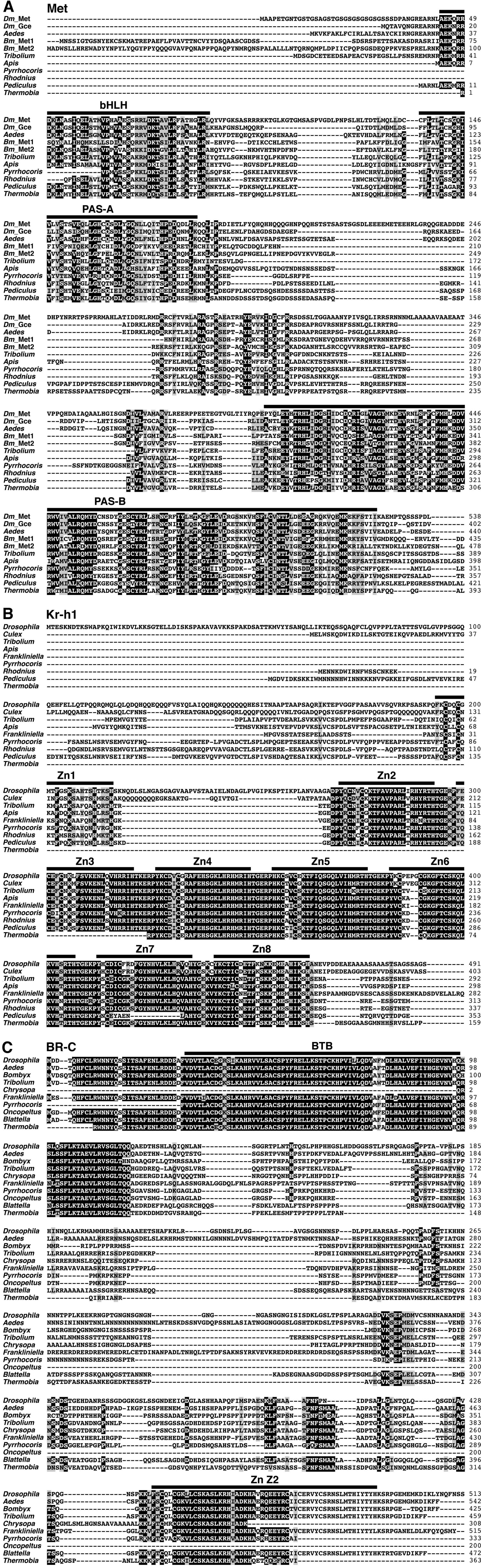
A
Met
bHLH
PAS-A
PAS-B
Dm_Met
Dm_Gce
Aedes
Bm_Met1
Bm_Met2
Tribolium
Apis
Pyrrhocoris
Rhodnius
Pediculus
Thermobia
B
Kr-h1
Zn1
Zn2
Zn3
Zn4
Zn5
Zn6
Zn7
Zn8
Drosophila
Culex
Tribolium
Apis
Frankliniella
Pyrrhocoris
Rhodnius
Pediculus
Thermobia
C
BR-C
BTB
Zn Z2
Drosophila
Aedes
Bombyx
Tribolium
Chrysopa
Frankliniella
Pyrrhocoris
Oncopeltus
Blattella
Thermobia
